# Stigma, stoma acceptance, quality of life among patients with colorectal cancer intestinal ostomy: a cross-sectional study

**DOI:** 10.3389/fonc.2026.1882299

**Published:** 2026-07-22

**Authors:** Xiaocui Zou, Li Xiao, Ling Hu, XiaoJuan Yang, Liqin Liang, Qing Wen, WenQing Guo, AiXin Zhou, Hua Zhang, Hong Wu

**Affiliations:** 1 Department of Hepatobiliary and Pancreatic Surgery, Sichuan Provincial People’s Hospital, University of Electronic Science and Technology of China, Chengdu, Sichuan, China; 2 Department of Geriatric Cardiology, Sichuan Provincial People’s Hospital, University of Electronic Science and Technology of China, Chengdu, Sichuan, China; 3 Department of Urology Surgery, Sichuan Provincial People’s Hospital, University of Electronic Science and Technology of China, Chengdu, Sichuan, China; 4 Department of Gastrointestinal Surgery, Sichuan Provincial People’s Hospital, University of Electronic Science and Technology of China, Chengdu, Sichuan, China; 5 Department of Nursing Department, Sichuan Provincial People’s Hospital, University of Electronic Science and Technology of China, Chengdu, Sichuan, China; 6 Department of Gastrointestinal Surgery, The First Affiliated Hospital of Chongqing Medical University, Yuanjiagang, Chongqing, China; 7 Department of Nursing Department,The First Affiliated Hospital of Chongqing Medical University, Yuanjiagang, Chongqing, China

**Keywords:** intestinal ostomy, latent profile analysis, mediation analysis, quality of life, stigma, stoma acceptance

## Abstract

**Background:**

Stigma is a common psychological distress among patients with colorectal cancer and an intestinal ostomy. It severely impacts their quality of life. However, the heterogeneity of stigma and its underlying mechanisms remain underexplored.

**Objective:**

This study aimed to identify latent subtypes of stigma in this population using latent profile analysis. It also examined factors associated with different stigma profiles and tested the mediating role of stoma acceptance between stigma profiles and quality of life.

**Methods:**

A cross-sectional survey was conducted among 449 patients with colorectal cancer and an intestinal ostomy recruited from tertiary hospitals in the Sichuan-Chongqing region, China. Stigma, stoma acceptance, and quality of life were assessed using the Social Impact Scale, Chinese version of the Stoma Acceptance Questionnaire, and Stoma-Quality of Life Questionnaire, respectively. Latent profile analysis was performed to identify stigma subtypes. Multinomial logistic regression examined predictors of profile membership. Mediation analysis tested whether stoma acceptance mediated the relationship between stigma profiles and quality of life.

**Results:**

Three distinct stigma profiles were identified: low (46.55%), moderate (39.20%), and high stigma groups (14.25%). Multivariate analysis revealed that being unmarried/divorced/widowed (OR = 2.28, 95% CI: 1.14-4.57); lower monthly household income (<3000 CNY) (OR = 8.65, 95% CI: 1.84-40.67); and complete dependence on others for stoma care (OR = 2.09, 95% CI: 0.97-4.51) were independent predictors of high stigma group membership. Significant stepwise decreases in stoma acceptance and quality of life scores were observed across the three profiles (all P < 0.05). Stoma acceptance partially mediated the relationship between stigma profiles and quality of life, with indirect effects accounting for 23.8% and 25.9% of the total effects in the moderate and high stigma groups, respectively.

**Conclusions:**

The characteristics of stigma among patients with colorectal cancer and intestinal ostomy. Stoma acceptance plays a significant mediating role in the relationship between stigma and quality of life.

**Clinical Trial Registration:**

http://www.chictr.org.cn/, identifier ChiCTR2500095532.

## Background

Colorectal cancer (CRC) is one of the most common malignancies worldwide and poses a serious threat to human health. According to the International Agency for Research on Cancer (IARC), CRC ranks third in incidence and second in mortality among all cancers globally. In 2020, approximately 1.93 million new cases and 0.9 million deaths were reported, with developed countries bearing the heaviest disease burden (1). In China, statistics from 2022 revealed a substantial burden of CRC, with 517,000 new cases and 240,000 deaths (2). These figures position CRC as the second most common cancer in terms of incidence and the fourth leading cause of cancer-related death in China (3). Despite continuous advances in surgical techniques and treatment modalities, intestinal ostomy surgery remains a primary treatment for many patients with low rectal cancer (4). It is estimated that the number of patients with an ostomy in China increases by more than 100,000 annually, and this trend is expected to continue growing (5). Although ostomy surgery can save and prolong life, it alters the patient’s excretory pathway, leading to involuntary defecation. Patients 1 often experience anxiety and feelings of inferiority due to issues such as a full stoma bag, irregular defecation, and altered body image, which severely impact their quality of life (6, 7).

Stigma, as a form of psychological stress response, often arises from patients’ perceived loss of identity and social status due to physical dysfunction. This sense of loss profoundly affects their daily life and psychological state and can trigger intense feelings of shame (8). Changes in excretory patterns, potential noise or odor from the stoma bag, and altered body image frequently expose patients to perceived social prejudice and discrimination. This leads to shame, isolation, and self-deprecation. Interview-based studies have found that patients with high levels of stigma often show reduced social motivation, diminished self-worth, and heightened insecurity, which may further result in social withdrawal (9, 10). Survey-based studies with larger samples have also shown that stigma levels among rectal cancer patients with anostomy are moderately high (11, 12).

These studies provide preliminary evidence for the relationship between stigma and psychosocial variables. However, a closer look at their analytical strategies reveals a commonly overlooked issue. Most previous studies have treated stigma as a homogeneous construct, using variable-centered approaches (such as correlation and regression analyses) to examine its relationships with other variables through total or domain scores (13–15). This analytical approach implicitly assumes that all patients share a uniform experience of stigma. For instance, Yuan et al. (16) and Xi et al. (17) analyzed the associations among stigma, stoma acceptance, and psychosocial adaptation based on total scale scores. Li et al. (18) tested the mediating role of stoma acceptance in the relationship between stigma and social isolation. However, none of these studies examined whether these relationships or mediating pathways differ across patient subgroups. Although variable-centered analyses are valuable, they may obscure clinically meaningful individual heterogeneity.

This heterogeneity suggests that a person-centered approach may provide a more detailed understanding of latent subgroup structures within the population and thus help identify patients with different risk profiles. Latent profile analysis (LPA) is one such method. It identifies latent subgroups based on individuals’ response patterns across observed indicators. LPA can more accurately capture population heterogeneity and aligns well with the clinical concept of “classified management” (19). Applying this method to study stigma among CRC patients with an intestinal ostomy may reveal distinct stigma subtypes and further identify their distinguishing characteristics—particularly modifiable factors—which could inform more precise clinical nursing.

Identifying subtypes, however, is only the first step. More importantly, we need to understand the pathways that underlie differences in quality of life across subtypes. Previous studies have established that stoma acceptance is positively correlated with quality of life (18, 20) and negatively correlated with stigma (16, 17). Some research has also examined the mediating role of stoma acceptance between stigma and quality of life (18). However, these mediation analyses remain at the variable level. They have not addressed a more specific question: whether the mediating role of stoma acceptance still holds across different stigma subtypes. This question remains unanswered.

To address this question, the present study adopts Folkman’s stress-appraisal-coping theory as its framework (21). This theory conceptualizes the psychological adaptation process following stressful events as three progressive stages: cognitive appraisal of the stressful situation, mobilization and implementation of coping strategies, and eventual adaptation outcomes. In the context of our study, stigma can be viewed as a negative cognitive appraisal—patients perceiving a threat to their identity and social status due to changes in physical function and appearance (10). Stoma acceptance represents a positive coping strategy through which patients achieve emotional acceptance of the stoma via cognitive restructuring (16). Quality of life serves as the ultimate adaptation outcome (22). The theory suggests that positive coping (acceptance) may buffer the impact of negative appraisal (stigma) on adaptation outcomes and that this process may differ across groups with varying stigma levels.

**Figure 1:**
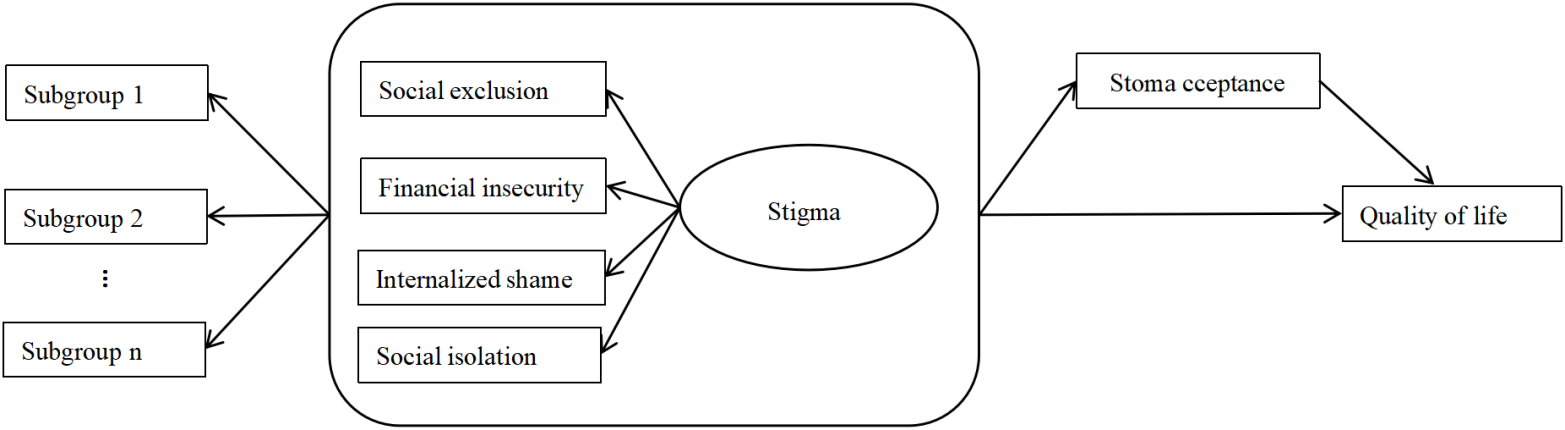
The hypothetical framework of stigma, acceptance, and quality of life among colorectal cancer and intestinal ostomy.

Based on the above theoretical and empirical foundations, this study pursues three main objectives. First, we use LPA to identify distinct stigma profiles among CRC patients with an intestinal ostomy so as to describe the heterogeneity of stigma experiences in this population more precisely. Second, we examine the predictive role of sociodemographic and clinical characteristics on profile membership, aiming to provide reference information for identifying high-risk groups. Third, within the stress-appraisal-coping framework, we test whether stoma acceptance shows a statistical pattern consistent with a mediating role between stigma profiles and quality of life. To our knowledge, this integrated analytical approach has rarely been systematically applied in this population. Accordingly, we propose the following hypotheses (**Figure 1**): (1) multiple distinct stigma profiles exist among CRC patients with an intestinal ostomy; and (2) stoma acceptance is consistent with a mediating role between these stigma profiles and quality of life.

## Methods

### Participants

An exploratory cross-sectional study was conducted in the Sichuan-Chongqing region of China between February and December 2025. Participants were recruited via convenience sampling from the gastrointestinal surgery departments of Class A tertiary-level hospitals in Chengdu and Chongqing. A review of previous LPA studies suggests that a sample size of at least 300 to 500 individuals is recommended to ensure stable and replicable solutions (23). Considering a 20% non-response rate, 500 participants were initially enrolled. The inclusion criteria were: (a) meeting the diagnostic criteria for CRC with a pathologically confirmed diagnosis (24); (b) undergoing initial ostomy surgery within 1 month; (c) being aged 18 years or over; and (d) voluntarily participating with signed informed consent. Exclusion criteria: (a) Severe psychiatric disorders or cognitive impairment; (b) Concurrent primary tumors in other sites; (c) Receiving active chemotherapy or radiotherapy at the time of enrollment. A total of 449 valid questionnaires were finally included, yielding a valid response rate of 89.8%.

### Procedures

Three nurses with research backgrounds and over five years of experience in clinical practice were selected from within the department to serve as investigators. The project leader provided standardized training to all three investigators. Before initiating the survey, the investigators were required to explain the study thoroughly to participants, obtain their consent, and have them sign informed consent forms. They then used standardized guidance language to explain the study’s purpose, content, significance, and key considerations for completing the questionnaire. While participants were completing the questionnaire, the investigators had to address any questions while avoiding leading language promptly. Once the questionnaires had been completed, the investigators collected them on-site and verified their completeness and the logical consistency of the items. Participants should then be asked promptly to supplement or correct any errors, omissions, or inconsistencies. This study has been approved by the hospital ethics committee (Approval No. 2022-409) and adheres to the principles outlined in the Declaration of Helsinki.

### Measures

#### Demographics characteristics

Our team independently designed the sociodemographic survey questionnaire based on a systematic review of relevant domestic and international literature. It includes questions on gender, age, educational attainment, marital status, per capita monthly household income, occupation, type of health insurance, participation in ostomy support meetings, self-care level of stoma management, surgical approach, type of stoma, ostomy subtype, and any stoma complications. Patients complete the questionnaire independently, and it is verified against electronic medical records.

#### Measurement of stigmatization

The Social Impact Scale (SIS) was developed by Fife et al. to measure the level of stigma experienced by patients with chronic diseases, such as cancer (25). This study used the Chinese version of the SIS, which was translated by Pan et al. (26). The scale comprises four dimensions—social exclusion, financial insecurity, internalized shame, and social isolation—and contains a total of 24 items. It uses a 4-point Likert scale, with 4 representing “strongly agree” and 1 representing “strongly disagree.” All items are reverse-scored, yielding a total score ranging from 24 to 96 points. Higher scores indicate greater levels of stigmatization. The Cronbach’s α for the scale was 0.92 (26). In this study, it was 0.965.

#### Measurement of stoma acceptance

The Chinese version of the Stoma Acceptance Questionnaire (SAQ-C), originally developed by Bagnasco et al. and subsequently translated and culturally adapted by Hu Ting, was used to assess patients’ acceptance of their intestinal ostomy (27, 28). The questionnaire comprises 12 items across three dimensions: four items evaluate importance, five assess autonomy and acceptance, and three assess evaluation support. It employs a 4-point Likert scale. For the importance dimension, responses range from 1 (“not at all important”) to 4 (“very important”). For autonomy and acceptance, they range from 1 (“not at all good”) to 4 (“very good”). For evaluation support, responses range from 1 (“strongly disagree”) to 4 (“strongly agree”). The total score ranges from 12 to 48 points, with higher scores indicating greater acceptance of the stoma. The scale has demonstrated good reliability in prior studies involving ostomy patients, with a reported Cronbach’s α of 0.826 (28). In this study, the Cronbach’s α for this scale was 0.951.

#### Measurement of quality of life

The Stoma-Quality of Life (Stoma-QOL) questionnaire was developed by Prieto et al. to comprehensively and sensitively measure the quality of life of patients with a colostomy or ileostomy, based on Hunt and McKenna’s basic QOL demand model (29). This study used the Chinese version of the Stoma-QOL (Stoma-QOL-C), which was translated and culturally adapted by Shao et al. (30). The scale comprises four dimensions—social relationship, psychological impact, defecation concern, and daily function — and contains a total of 20 items. It uses a 4-point Likert scale, with 1 representing “always,” 2 representing “sometimes,” 3 representing “rarely,” and 4 representing “never.” The total score is calculated by summing the scores of all items, with a possible range of 20 to 80 points. The Cronbach’s α coefficient for the Stoma-QOL-C was 0.935 (30). In this study, the Cronbach’s α for the scale was 0.949.

### Data analyses

Data were analyzed using SPSS 22.0, Mplus 8.3, and JASP 0.16.0. Statistical significance was set at *P* < 0.05. Because data were collected through self-reported questionnaires at a single time point, Harman’s single-factor test was used as an initial check for common method bias. The first unrotated factor explained 40. 10% of the total variance, which is slightly above the conventional 40% threshold. While this suggests that common method bias may not be a severe concern, we acknowledge that Harman’s test alone is a limited method for detecting common method variance.

First, descriptive statistics were calculated. Categorical variables were described using frequencies and percentages. Continuous variables were presented as means and standard deviations. Differences between subgroups were examined using independent samples t-tests, analysis of variance (ANOVA), or Mann–Whitney U tests, as appropriate.

Second, bivariate associations among stigma, stoma acceptance, and quality of life were examined using Pearson correlation analysis.

Third, all 24 individual items of the Social Impact Scale (SIS) were entered as indicators for the LPA. The SIS covers four dimensions (social exclusion, financial insecurity, internalized shame, and social isolation), but we retained the item−level data to capture fine−grained response patterns that may reveal heterogeneity beyond dimension−level aggregates. LPA was conducted in Mplus 8.3. The default model specification was adopted, which assumes that variances are equal across classes and covariances among indicators are fixed at zero. This is the most commonly used model specification in LPA, as it ensures model identification while avoiding overparameterization. All four stigma dimensions were measured on the same 4-point Likert scale, with identical response anchors. Therefore, raw subscale scores were used as LPA indicators without standardization to preserve interpretability of the latent profiles on the original metric. To ensure robust latent profiles, sensitivity analyses included re-running the LPA with different starting values and examining outliers. To avoid convergence to local maxima, we used 500 random sets of starting values with 100 final stage optimizations and the AUTO option in Mplus. The best log-likelihood value was replicated across multiple random starts, suggesting convergence to a global maximum rather than a local solution. Models with one to five latent classes were estimated and compared. Fit indices used were AIC, BIC, and sample-size-adjusted BIC (aBIC); lower values indicated better fit. The LMR and BLRT tests were also applied. A significant p-value (*P* < 0.05) meant the k-class model fit better than the (k- 1)-class model. Classification accuracy was evaluated with entropy. Values closer to 1 (≥ 0.8, indicating ≥ 90% accuracy) suggested clearer profile delineation. The selected model balanced goodness of fit, parsimony, and interpretability (31, 32). Factors associated with LPA-based stigma were examined using univariable and multivariable logistic regression analyses (33).

Fourth, differences in stoma acceptance and quality of life scores across the LPA-based stigma were compared using ANOVA and *post-hoc* analysis. *Post hoc* pairwise comparisons following ANOVA were conducted using a Bonferroni correction to control for family-wise Type I error across multiple simultaneous comparisons.

Finally, to examine whether stoma acceptance shows a statistical pattern consistent with a mediating role between stigma profiles and quality of life, mediation analysis was performed using the PROCESS macro (Model 4) with 5,000 bootstrap resamples.

### Ethical considerations

Our study was conducted in accordance with the principles outlined in the Declaration of Helsinki. All the procedures performed in this study were approved by the Medical Ethics Committee of Sichuan Provincial Academy of Medical Sciences-Sichuan Provincial People’s Hospital (Approval No. 2022-409). All participants provided written informed consent for the use of their data in non-commercial scientific research. All participants were informed of their right to withdraw from the study at any time without negative consequences.

## Results

### Demographic and clinical characteristics of participants with stigma scores stratified by each variable

A total of 449 patients with CRC and intestinal ostomies participated in this study. The mean age at the time of the survey was 64.04 years (SD = 11.728), ranging from 19 to 95 years. The sample comprised 299 males (66.6%) and 150 females (33.4%). Regarding educational attainment, 27.2% had primary school or below, 27.8% had middle school, 29.6% had high school, and 15.4% had college or above. Most participants were married (59.5%), and 40.5% were unmarried/divorced/widowed. The per capita monthly household income was distributed as follows: less than 3,000 CNY (31.4%), 3,000~4,999 CNY (44.8%), or 5,000 CNY or more (23.8%). Occupation categories included employed (55.5%), unemployed (16.5%), and retired (28. 1%). The types of health insurance varied, with the largest groups being New Rural Cooperative (37.0%) and Urban & Rural Residents (33.0%).

Regarding stoma-related characteristics, 215 participants (47.88%) reported having participated in ostomy support meetings, while 234 (52. 12%) had not. With respect to the self-care level of stoma management, 22.0% of patients were completely independent, 47.0% were partially independent, and 31.0% were completely dependent. In addition, most patients underwent laparoscopic surgery (88.2%), had a temporary stoma (76.0%), and had no stoma complications (86.2%). The type of stoma was nearly equally distributed between colostomy (48.3%) and ileostomy (51.7%).

Turning to the relationship between demographic and clinical variables and perceived stigma, univariate analyses revealed that higher stigma scores were significantly associated with lower educational attainment (primary school or below), unmarried/divorced/widowed marital status, lower per capita monthly household income (<3000 CNY), complete dependence on others for stoma management, and the presence of stoma complications (all *P* < 0.05). In contrast, no significant differences in stigma scores were found for gender, occupation, type of health insurance, participation in ostomy support meetings, surgical approach, type of stoma, or ostomy subtype, as shown in **Table 1**.

**Table 1:** Demographic and clinical characteristics of participants with stigma scores stratified by each variable (N = 449).

Variable	Category	n (%)	Stigma score (M ± SD)	Statistic	*P*-value
Gender	Male	299 (66.59)	64.01 ± 12.91	-1.053a	0.293
	Female	150 (33.41)	65.36 ± 12.72		
Age (years)	≤ 60	175 (38.98)	63.71 ± 12.44	0.981a	0.327
	> 60	274 (61.02)	64.93 ± 13.10		
Educational attainment	Primary school or below	122 (27.17)	69.12 ± 15.32	16.066c	**0.001**
	Junior high school	125 (27.84)	62.94 ± 12.14		
	High school/Technical secondary school	133 (29.62)	63.27 ± 10.48		
	College or above	69 (15.37)	61.25 ± 11.46		
Marital status	Married	267 (59.47)	62.52 ± 11.15	-3.043c	**0.002**
	Unmarried/Divorced/Widowed	182 (40.53)	67.30 ± 14.57		
Per capita monthly household income (CNY)	< 3000	141 (31.40)	66.93 ± 13.51	16.340c	**<0.001**
	3000–4999	201 (44.77)	65.14 ± 13.31	
	≥ 5000	107 (23.83)	59.93 ± 9.61		
Occupation	Employed	249 (55.46)	65.24 ± 12.77	1.026b	0.359
	Unemployed	74 (16.48)	63.51 ± 12.97		
	Retired	126 (28.06)	63.48 ± 12.93		
Type of health insurance	Self-funded	37 (8.24)	64.51 ± 13.71	1.692b	0.168
	New Rural Cooperative Medical Scheme	166 (36.97)	65.51 ± 12.99		
	Urban-Rural Resident Basic Medical Insurance	148 (32.96)	64.93 ± 12.99		
	Employee Medical Insurance	98 (21.83)	61.95 ± 11.88		
Participation in ostomy support meetings	No	234 (52.12)	65.12 ± 13.15	1.13a	0.259
	Yes	215 (47.88)	63.74 ± 12.50		
Self-care level of stoma management	Complete self-care	99 (22.05)	64.82 ± 11.97	15.447c	**<0.001**
	Partial self-care	211 (46.99)	61.71 ± 10.79		
	Complete dependence on others	139 (30.96)	68.38 ± 15.15		
Surgical approach	Open surgery	53 (11.80)	66.98 ± 13.47	1.524a	0.128
	Laparoscopic surgery	396 (88.20)	64.12 ± 12.74		
Type of stoma	Temporary ostomy	341 (75.95)	63.94 ± 12.50	-1.528a	0.127
	Permanent ostomy	108 (24.05)	66.10 ± 13.80		
Ostomy subtype	Colostomy	217 (48.33)	64.37 ± 13.14	-0.144a	0.886
	Ileostomy	232 (51.67)	64.54 ± 12.59		
Stoma complications	No	387 (86.19)	63.63 ± 11.94	-2.247c	**0.0247**
	Yes	62 (13.81)	69.65 ± 16.69		

a: t-value; b: F-value; c: H-value. Univariate analysis was performed using independent t-tests for normally distributed data with homogeneity of variance between two groups, ANOVA for multiple groups, Mann-Whitney U tests for non-normally distributed data between two groups, and Kruskal-Wallis tests for multiple groups with non-normal distribution. Bold figures indicate statistically significant results (*P* < 0.05).

### Correlation analysis of stigma, stoma acceptance, and quality of life

**Figure 2:**
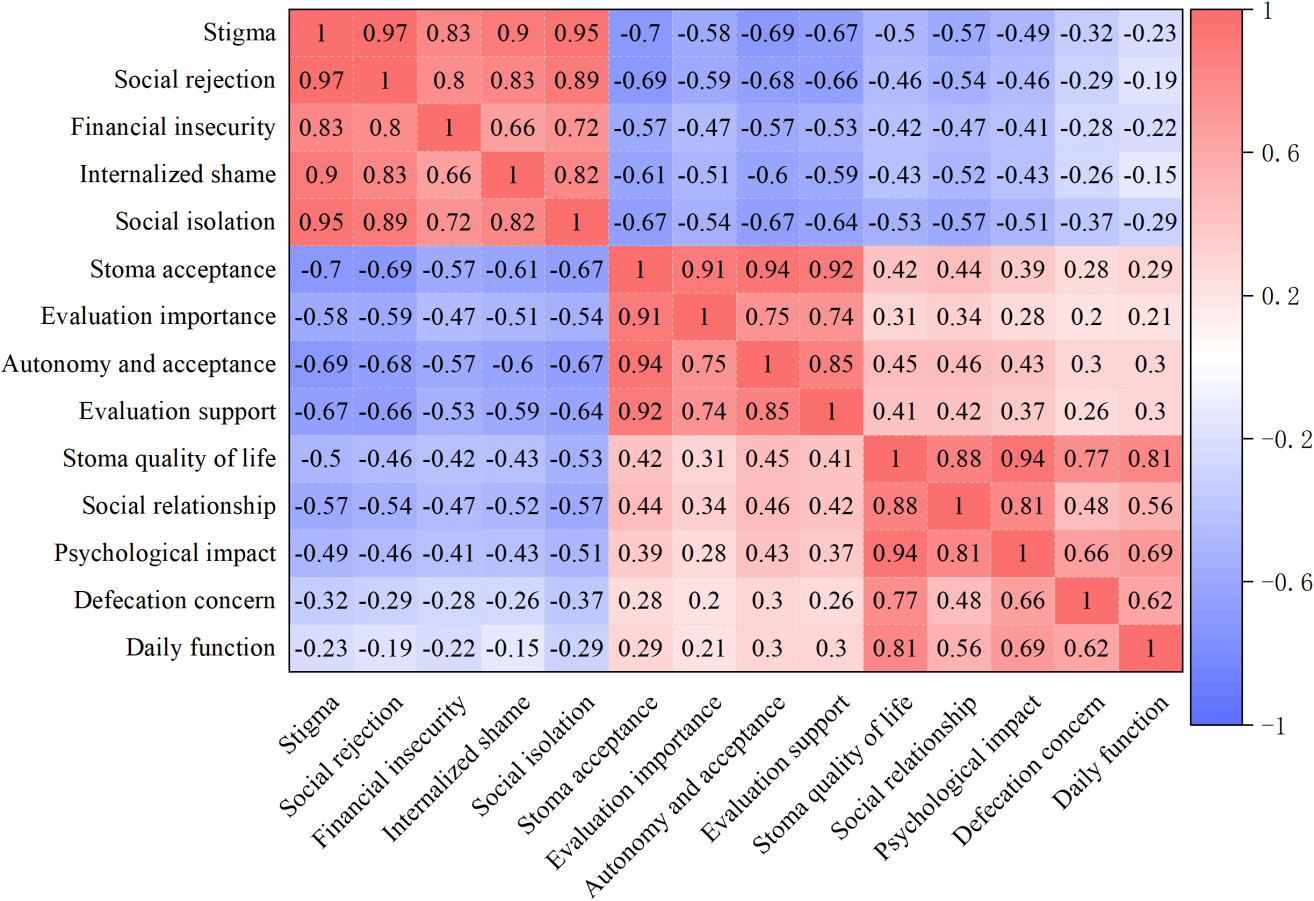
Pearson correlation heatmap of stigma, acceptance, and quality of life. C1 = Low stigma group, C2 = Moderate stigma group, C3 = High stigma group.

The mean and standard deviations of variables were stigma (64.46 ± 12.85), stoma acceptance (32.13 ± 7.78), and stoma quality of life (46.06 ± 11.83). **Figure 2** illustrates Pearson’s correlation heatmap (all *P* < 0.001). Stigma was negatively associated with stoma acceptance (r = −0.70) and stoma quality of life (r = −0.50). Stoma acceptance was positively associated with stoma quality of life (r = 0.42). These significant correlations provided a foundation for subsequent mediation analysis.

### Latent profiles analysis of stigma

**Figure 3:**
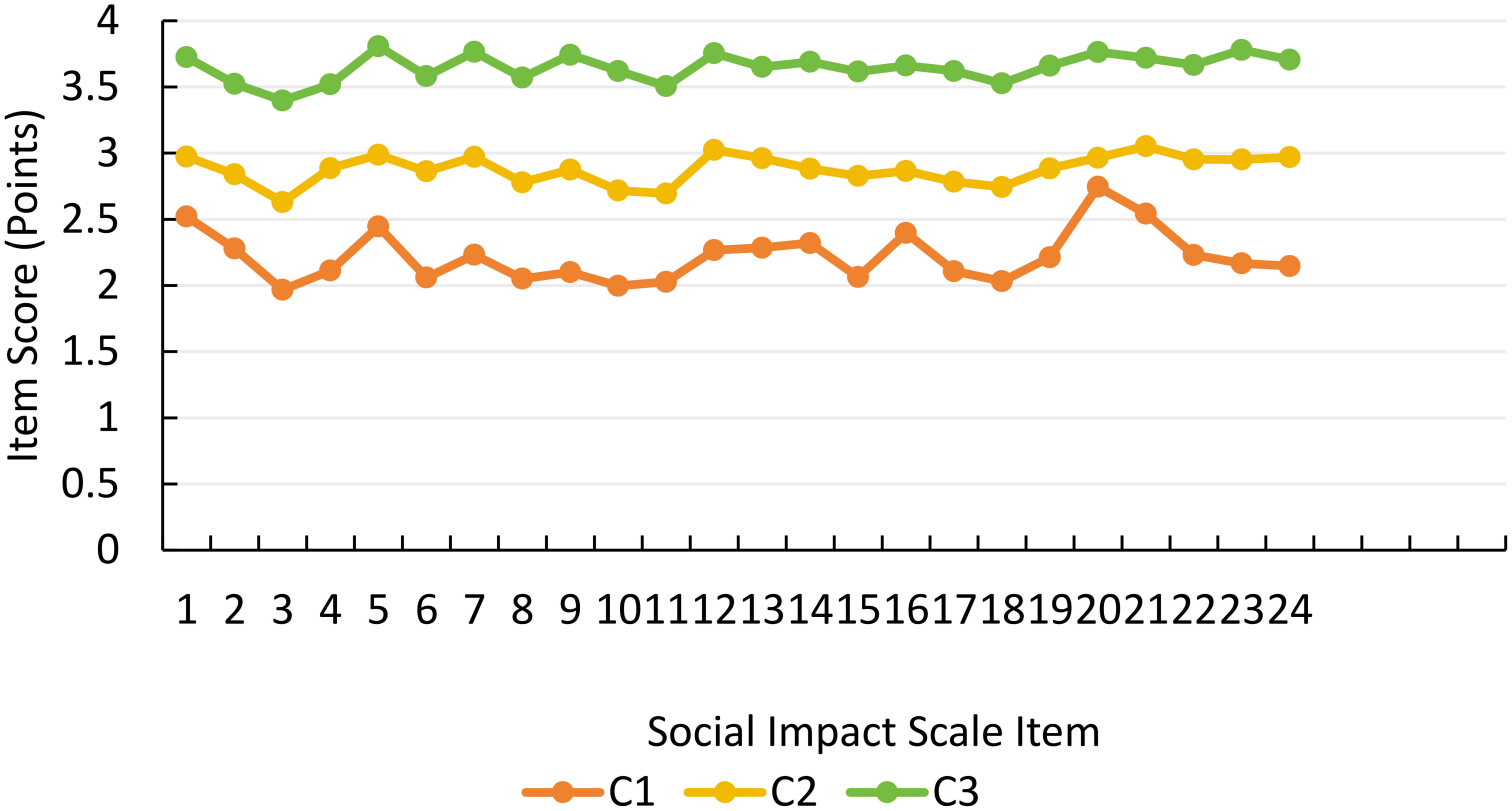
Latent profile characteristics of stigma (3-profile solution).

Although fit indices suggested potential improvement with more classes (e.g., lower AIC/BIC values for 4-class and 5-class solutions), we selected the 3-class solution based on the following considerations: (a) interpretability—the three classes displayed a clear low-moderate-high gradient pattern; (b) parsimony—the 3-class solution provides a simpler and more clinically actionable framework compared to solutions with more classes; (c) class size—the smallest class in the 3-class solution (Class 3, n=64, 14.25%) exceeds the recommended minimum sample size of 5% of the total sample or 30–50 cases per class; (d) entropy—the 3-class solution had the highest entropy (0.972) among all solutions, indicating superior classification clarity; and (e) the LMR-LRT was significant for the 3-class solution (*P* < 0.001) but non-significant for the 4-class solution (*P* = 0.215), suggesting that adding a fourth class did not significantly improve model fit. The best models were found to be of three types based on theory, parsimony, and fitting indicators. **Table 2** and **Figure 3** detail additional information. Class 1 (low stigma group, 46.55%) exhibited consistently low scores across all four dimensions (social exclusion: M = 19.45, SD = 1.75; financial insecurity: M = 6.91, SD = 1.24; internalized shame: M = 11.12, SD = 1.60; social isolation: M = 15.83, SD = 1.73). Class 2 (moderate stigma group, 39.20%) showed moderate elevations across all dimensions (Ms = 8.71-25.74). Class 3 (high stigma group, 14.25%) displayed marked elevations across all dimensions (Ms = 10.80-32.80), with social exclusion being the most pronounced dimension (M = 32.80, SD = 2.24). Hypothesis 1 was supported.

**Table 2:** Fit indices for latent profile analysis of stigma.

Indications			LPA model		
	1-Class	2-Class	3-Class	4-Class	5-Class
Fit statistics					
AIC	23587.888	18817.752	**17100.089**	16688.896	16439.867
BIC	23785.02	19117.564	**17502.577**	17194.06	17047.706
aBIC	23632.692	18885.891	**17191.563**	16803.706	16578.012
Entropy	—	0.971	**0.972**	0.952	0.94
LMRT	—	<0.001	**<0.001**	0.2147	0.0879
BLTR	—	<0.001	**<0.001**	<0.001	<0.001
Group size (%)					
C1	100	62.14	**46.55**	41.87	19.15
C2		37.86	**39.2**	23.61	23.61
C3			**14.25**	23.39	23.16
C4				23.39	21.6
C5					12.47

AIC, Akaike Information Criterion; BIC, Bayesian Information Criterion; aBIC, sample-size adjusted BIC; LMRT, Lo–Mendell–Rubin adjusted likelihood ratio test; BLRT, bootstrap likelihood ratio test.

Bold figures highlight the selected class solution (3-class).

Additionally, the average posterior probabilities (APP) for the three categories are reported in the text as 0.9911, 0.981, and 0.998, respectively. These results significantly exceed the acceptable threshold of 0.70, demonstrating excellent classification accuracy.

Using multivariable logistic regression analyses (see **Table 3**), factors associated with latent class membership were examined, with the low-stigma group as the reference. In multivariate analysis, being married was a risk factor for the high-stigma group (OR = 2.28, 95% CI: 1.14–4.57, *P* = 0.020). Lower per capita monthly household income was also a risk factor, both for the < 3000 CNY group (OR = 8.65, 95% CI: 1.84–40.67, *P* = 0.006) and the 3000–4999 CNY group (OR = 6.38, 95% CI: 1.36–29.85, *P* = 0.019). Regarding stoma self-care, partial independence was a protective factor (OR = 0.38, 95% CI: 0.18–0.80, *P* = 0.011), indicating that patients who were completely dependent on others had higher odds of high stigma. No significant predictors were identified between the moderate-stigma and low-stigma groups.

**Table 3:** Univariate and multivariate logistic regression results for predicting external features of the three-class pattern.

Variables		Univariate analysis			Multivariate analysis	
	C2vsC1	C3vsC1	*P*	C2vsC1	C3vsC1	
	OR(95%CI)	*P* OR(95%CI)		OR(95%CI)	*P* OR(95%CI)	*P*
Educational attainment (ref. College or above)						
Primary school or below	1.10(0.58-2. 1)	0.770 6.55(2. 14-20. 11)	**0.001**	1.00(0.48-2. 11)	0.993 2.29(0.66-7.94)	0.193
Middle school	0.94(0.51- 1.76)	0.856 2.50(0.78-7.98)	0.123	0.87(0.45- 1.69)	0.689 1.73(0.50-6.04)	0.390
High school	1.37(0.75-2.5)	0.313 1.37(0.39-4.75)	0.625	1.30(0.70-2.40)	0.411 1.47(0.39-5.48)	0.569
Marital status (ref.Unmarried/divorced/widowed))						
Married	0.95(0.63- 1.45)	0.817 3.85(2. 12-7.01)	**<0.001**	1.00(0.63- 1.58)	0.987 2.28(1. 14-4.57)	**0.020**
Per capita monthly household income(ref. ≥5000 CNY)						
<3000CNY	1.53(0.89-2.61)	0.123 15.75(3.59-69. 12)	**<0.001**	1.66(0.93-2.96)	0.089 8.65(1.84-40.67)	**0.006**
3000∼4999CNY	1.28(0.78-2. 11)	0.323 11.9(2.76-51.34)	**0.001**	1.51(0.87-2.62)	0.143 6.38(1.36-29.85)	**0.019**
Self-care level of stoma management (ref. Completely dependent)						
Completely independent	1.32(0.74-2.34)	0.346 0.38(0.17-0.84)	**0.016**	1.32(0.68-2.54)	0.412 1.26(0.49-3.22)	0.635
Partially independent	0.83(0.51- 1.34)	0.439 0.19(0.10-0.37)	**<0.001**	0.80(0.48- 1.36)	0.415 0.38(0.18-0.8)	**0.011**

Only variables that showed statistically significant associations (*P* < 0.05) in either univariate or multivariate analyses are presented in this table. Non-significant variables were omitted for clarity and conciseness. Bold figures indicate statistically significant results (*P* < 0.05).

C1, low stigma group; C2, moderate stigma group; C3, high stigma group; OR, odds ratio; CI, confidence interval.

These findings suggest that being married, having a low income, and complete dependence on others for stoma care are important determinants of membership in the high-stigma group.

### LPA-based stigma differences on stoma acceptance and quality of life scores

As shown in **Table 4**, significant differences across the three stigma profiles were observed in both stoma acceptance and stoma quality of life scores.

**Table 4:** ANOVA comparisons of stoma acceptance and quality of life scores across LPA-based stigma profiles and *post-hoc* comparisons.

Variables	C1 (n=209) (M ± SD)	C2 (n=176) (M ± SD)	C3 (n=64) (M ± SD)	*F*	*P*	*Post-hoc*
**Stoma acceptance**	36.24 ± 4.16	30.95 ± 6.70	21.94 ± 9. 12	139.201	**<0.001**	1 > C2 > C3
Evaluation importance	12.46 ± 1.84	10.68 ± 2.94	7.75 ± 3.67	82.581	**<0.001**	C1 > C2 > C3
Autonomy and acceptance	14.81 ± 1.99	12.64 ± 2.79	8.78 ± 3.64	135.666	**<0.001**	C1 > C2 > C3
Evaluation support	8.98 ± 1.21	7.63 ± 1.83	5.41 ± 2.42	115.738	**<0.001**	C1 > C2 > C3
**Stoma quality of life**	50.46 ± 9.71	44.72 ± 10.87	35.39 ± 13.06	50.851	**<0.001**	C1 > C2 > C3
Social relationship	17.38 ± 3.22	13.98 ± 3.98	11. 11 ± 4.85	80.88	**<0.001**	C1 > C2 > C3
Psychological impact	15.46 ± 3.06	13.49 ± 3.60	10.67 ± 4.15	50.607	**<0.001**	C1 > C2 > C3
Defecation concern	8.90 ± 2.64	8.39 ± 2.44	6.56 ± 2.25	21.274	**<0.001**	C1 > C2 > C3
Daily function	8.71 ± 3.15	8.86 ± 2.44	7.05 ± 2.89	10.226	**<0.001**	C1, C2 > C3

C1 = Low stigma group, C2 = Moderate stigma group, C3 = High stigma group. *Post hoc* comparisons were conducted using Bonferroni-corrected tests. Bold figures indicate statistically significant results (*P* < 0.05).

For stoma acceptance, significant between-group differences were found for the total score (*F* = 139.201, *P* < 0.001) and all three subscales: evaluation importance (*F* = 82.581, *P* < 0.001), autonomy and acceptance (*F* = 135.666, *P* < 0.001), and evaluation support (*F* = 115.738, *P* < 0.001). *Post hoc* pairwise comparisons using Bonferroni correction revealed that stoma acceptance scores decreased progressively from the low stigma group to the moderate stigma group to the high stigma group(all *P* < 0.05), indicating a dose-response relationship between stigma level and acceptance.

For stoma quality of life, significant between-group differences were found for the total score (*F* = 50.851, *P* < 0.001) and all four subscales: social relationship (*F* = 80.88, *P* < 0.001), psychological impact (*F* = 50.607, *P* < 0.001), defecation concern (*F* = 21.274, *P* < 0.001), and daily function (*F* = 10.226, *P* < 0.001). *Post-hoc* comparisons revealed that for the total score and most subscales (social relationship, psychological impact, and defecation concern), scores decreased progressively from the low stigma group to the moderate stigma group to the high stigma group (all *P* < 0.05). For the daily function subscale, the low and moderate stigma groups both had significantly higher scores than the high stigma group (*P* < 0.05), but no significant difference was found between the low and moderate stigma groups (*P*>0.05).

These findings demonstrate that patients with different stigma profiles exhibit distinct patterns of stoma acceptance and quality of life, with higher stigma levels consistently associated with lower acceptance and poorer quality of life.

### Mediation analysis of stoma acceptance between LPA-based stigma and quality of life

**Figure 4:**
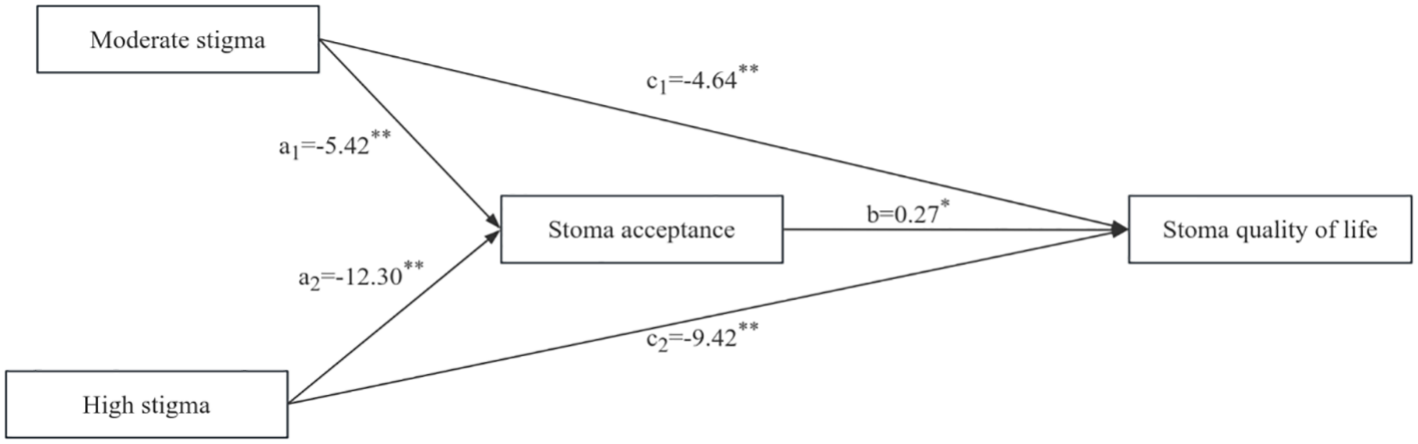
Hypothesized theoretical model. The three LPA-based stigma profiles are the independent variable (X), stoma acceptance is a mediator (M), and stoma quality of life is the dependent variable (Y). The low stigma group is used as the reference. The control variables are not presented in the image for brevity. ^∗ ∗^ *P* < 0.001, ^∗^ *P* < 0.01.

As shown in **Table 5**, **Table 6**, and **Figure 4**, stoma acceptance significantly mediated the relationship between stigma profiles and quality of life, with the low stigma group as reference. Demographic and clinical characteristics (educational attainment, marital status, per capita monthly household income, self-care level of stoma management, and stoma complications) were controlled as covariates.

**Table 5:** The mediating effect of stigma profiles on stoma quality of life.

Variables	*β*	SE	t	P	LLCI	ULCI	R²
Outcome variable: Stoma acceptance							0.459
Moderate stigma group	-5.422	0.593	-9.138	<0.001	-6.589	-4.256	
High stigma group	- 12.296	0.876	- 14.043	<0.001	- 14.017	- 10.575	
Educational attainment	1.162	0.315	3.688	<0.001	0.543	1.781	
Marital status	- 1.273	0.614	-2.074	0.039	-2.479	-0.067	
Per capita monthly household income	0.272	0.394	0.691	0.490	-0.503	1.048	
Self-care level of stoma management	- 1.059	0.427	-2.482	0.013	- 1.897	-0.220	
Stoma complications	- 1.341	0.837	- 1.601	0.110	-2.987	0.305	
Outcome variable: Stoma quality of life							0.262
Moderate stigma group	-4.638	1.150	-4.034	<0.001	-6.898	-2.379	
High stigma group	-9.416	1.872	-5.031	<0.001	- 13.094	-5.738	
Stoma acceptance	0.267	0.085	3.156	0.002	0.101	0.433	
Educational attainment	0.202	0.568	0.355	0.723	-0.915	1.318	
Marital status	- 1.844	1.096	- 1.683	0.093	-3.997	0.309	
Per capita monthly household income	-0.569	0.701	-0.812	0.417	- 1.948	0.809	
Self-care level of stoma management	-2.378	0.763	-3.116	0.002	-3.878	-0.878	
Stoma complications	-2.183	1.492	- 1.463	0. 144	-5.116	0.750	

B, unstandardized coefficient; SE, standard error; *t*, *t*-value; *P*, p-value; LLCI, lower limit of 95% confidence interval; ULCI, upper limit of 95% confidence interval; R², R-squared.

Low stigma group is used as the reference category for stigma profiles. All demographic and clinical.

characteristics (educational attainment, marital status, per capita monthly household income, self-care level of stoma management, and stoma complications) were entered as covariates.

**Table 6:** Direct and indirect effects of stigma profiles on stoma quality of life.

Variables	Effect	SE	LLCI	ULCI
Direct effect				
Moderate stigma group	-4.638	1.150	-6.898	-2.379
High stigma group	-9.416	1.872	- 13.094	-5.738
Indirect effect				
Moderate stigma group	- 1.448	0.502	-2.484	-0.513
High stigma group	-3.284	1.157	-5.639	- 1.147
Total effect				
Moderate stigma group	-6.086	1.065	-8.179	-3.993
High stigma group	- 12.700	1.571	- 15.788	-9.611

B, unstandardized coefficient; SE, standard error; *t*, t-value; *P*, p-value; LLCI, lower limit of 95% confidence interval; ULCI, upper limit of 95% confidence interval; R², R-squared.

Low stigma group is used as the reference category for stigma profiles. All demographic and clinical.

characteristics (educational attainment, marital status, per capita monthly household income, self-care level of stoma management, and stoma complications) were entered as covariates. Bootstrap resamples = 5,000.

Compared to the low stigma group, patients in the moderate stigma group (*β* = −5.422, *P* < 0.001) and high stigma group (*β* = −12.296, *P* < 0.001) reported significantly lower stoma acceptance, which in turn was positively associated with quality of life (*β* = 0.267, *P* = 0.002).

The indirect effects were significant for both the moderate stigma group (indirect effect = - 1.448, 95% CI: -2.484 to -0.513) and the high stigma group (indirect effect = -3.284, 95% CI: -5.639 to - 1. 147). The direct effects also remained significant (Moderate: -4.638, 95% CI: -6.898 to -2.379; High: -9.416, 95% CI: - 13.094 to -5.738), indicating partial mediation. The mediation effects accounted for 23.8% and 25.9% of the total effects in the moderate and high stigma groups, respectively. Thus, Hypothesis 2 was supported.

## Discussion

This study employed LPA to identify three distinct subtypes of stigma among patients with CRC and an intestinal ostomy: the low-stigma group (46.55%), the moderate-stigma group (39.20%), and the high-stigma group (14.25%), thus supporting Hypothesis 1. Compared to the low and moderate stigma groups, the high stigma group exhibited consistently elevated scores across all stigma dimensions. This finding indicates that these individuals not only perceive strong social exclusion and isolation but also experience profound internalized shame and self-deprecation, with their level of psychological distress far exceeding that of the other two groups. This result is consistent with the findings of Mi et al. (34). Although the proportion of individuals in the high stigma group was relatively small, they represent a critical population requiring urgent psychological intervention. Intense feelings of shame often lead them to actively avoid social situations and develop resistance to stoma care, thereby becoming trapped in a negative cycle where “stigma exacerbates social isolation, ultimately leading to a deterioration in quality of life” (35).

In this study, being married, having a low income, and complete dependence on others for stoma care were identified as risk factors for high stigma, whereas partial independence in stoma self-care was a protective factor. Regarding marital status, married patients paradoxically faced a higher risk of stigma, which differs from some previous findings (36). A point worth clarifying is that the univariate analysis showed higher mean stigma scores among unmarried/divorced/widowed participants. However, the multivariable LPA model identified being married as a risk factor for high-stigma class membership. This apparent discrepancy reflects the different analytical approaches used. The univariate analysis examines crude bivariate associations without adjustment for other variables. The multivariable model assesses the independent contribution of marital status after controlling for covariates such as income, education, and self-care ability. Being married thus carries a unique risk for belonging to the high-stigma subgroup. This risk is not captured by simple mean comparisons. A plausible explanation is that married patients may experience heightened stigma due to concerns about burdening their spouse. Body image issues within intimate relationships may also contribute. Guilt over impaired family role fulfillment may further intensify this experience (37). Regarding economic status, low income directly restricts access to high- quality stoma care supplies and psychological counseling services. It also increases the risk of leakage and odor, which exacerbates social concerns. At the same time, financial stress itself consumes psychological coping resources, creating a dose- response relationship (38). Regarding stoma self- care ability, complete dependence on others means patients lose autonomous control over their bodies. Their self- efficacy decreases, and they are more likely to view the stoma as a “defect” and internalize social prejudice, which significantly intensifies feelings of shame and inferiority (39). In contrast, patients who are partially independent retain some sense of control and self-efficacy, which helps buffer against stigma. Together, these vulnerability characteristics reveal the multidimensional psychosocial risks of the high- stigma group, providing clear targets for early clinical identification and stratified intervention.

The present study revealed a stepwise decrease in both total and domain scores of stoma acceptance, as well as in total quality of life and its domains (except for daily function), from the low to the high stigma group (all P < 0.05). This dose-response relationship provides robust evidence for the negative association between stigma and quality of life. As stigma levels increase, patients are more likely to adopt avoidant rather than approach-oriented coping strategies, characterized by a reluctance to learn self-care skills and an avoidance of social situations (38, 40, 41). This divergence in coping styles is directly reflected in the observed stepwise decline in stoma acceptance. Notably, for the daily function domain, both the low and moderate stigma groups scored significantly higher than the high stigma group, while no significant difference was found between the former two groups. This finding suggests that impaired daily function may be a “threshold characteristic” of the high stigma group—meaning that behavioral limitations manifest only when the accumulation of stigma reaches a critical psychological threshold (37). Clinical observations indicate that patients with high levels of stigma often voluntarily reduce their outdoor activities due to excessive worry about stoma bag leakage or odor, and this behavioral inhibition directly leads to restricted daily functioning (42).

Mediation analysis revealed that stoma acceptance is consistent with a partial mediating role in the relationship between stigma and quality of life. This finding supports Hypothesis 2 and clarifies the pathway through which stigma affects quality of life. High levels of stigma erode patients’ self-efficacy and hope, both of which serve as psychological drivers for transitioning from “resistance” to “acceptance” (34, 43). When patients are immersed in shame and self-deprecation, they face greater difficulty initiating cognitive restructuring and integrating the stoma into their self-concept, manifesting as reduced stoma acceptance (44). This diminished acceptance further impedes adaptive behaviors such as learning self-care skills, rebuilding social connections, and resuming daily activities, ultimately leading to a comprehensive decline in quality of life (45). The mediating effect accounted for 24% to 26% of the total effect, indicating that approximately one-quarter of stigma’s impact on quality of life is mediated through the pathway of eroding acceptance.

In summary, this study identified three latent subtypes of stigma among CRC patients with an intestinal ostomy through LPA, revealing the heterogeneous nature of stigma in this population. Additionally, mediation analysis revealed that stoma acceptance is consistent with a partial mediating role in the relationship between stigma and quality of life. Profound stigma severely erodes patients’ self-efficacy and hope levels, impairing their ability to engage in effective cognitive reconstruction, which behaviorally manifests as reduced stoma acceptance (46). This low level of acceptance further hinders adaptive behaviors, such as mastering self-care skills and rebuilding social connections, ultimately leading to an overall decline in their quality of life (16). According to the stress-appraisal-coping theory, stigma, as a negative cognitive appraisal, diminishes patients’ motivation and capacity to adopt positive coping strategies (47); conversely, stoma acceptance represents a positive cognitive restructuring strategy that can buffer the impact of negative appraisals on psychological adaptation (48); quality of life serves as the comprehensive adaptive outcome of these sequential psychological processes (49). Within the theoretical framework of stress-appraisal-coping, this statistical pattern suggests a potential mechanism; however, given the cross-sectional design, these findings should be interpreted as theoretically plausible rather than causally established.

### Clinical implications

Our findings generate several preliminary hypotheses for future research. For the low-stigma group, routine health education and regular monitoring may suffice to maintain adaptation. For the moderate-stigma group, targeted interventions—including cognitive-behavioral components, peer support, and self-efficacy enhancement—may help prevent progression to high stigma. For the high-stigma group, a multidisciplinary approach integrating psychological counseling, specialized ostomy nursing, family involvement, and mental health referral may be beneficial. However, these proposals are derived from associational cross-sectional data; their clinical utility and effectiveness require testing in future interventional studies before any practice recommendations can be made.

### Limitations

The present study has several limitations. First, the cross-sectional design precludes causal inference. Although our mediation findings are statistically consistent with the proposed model, temporal precedence cannot be established. Alternative model specifications—such as reverse causality or reciprocal relationships between stigma and acceptance—remain plausible and should be tested in future longitudinal studies. Second, all data were obtained via self-report questionnaires, raising the potential for common method bias. Although Harman’s single-factor test suggested that common method bias was not a major concern (the first unrotated factor explained 40. 10% of the variance), future studies should consider using multi-source data (e.g., clinician ratings or objective clinical indicators) to strengthen validity. Third, classification uncertainty in LPA-based analyses was not fully accounted for in the subsequent logistic regression and mediation models. However, the high entropy (0.972) and average posterior probabilities exceeding 0.98 for all classes suggest that classification uncertainty was minimal in our sample. Future studies with larger samples may apply the three-step approach to more rigorously handle classification error. Fourth, the sample was recruited from tertiary hospitals in the Sichuan-Chongqing region using convenience sampling, and the high-stigma group was relatively small, which may limit generalizability and statistical power. Future multicenter studies with larger samples are needed to validate our findings.

### Future directions

Future studies with larger samples are warranted to further validate the subgroup classification and the mediation model. Future research should address several important questions. First, longitudinal studies are needed to establish the temporal ordering among stigma, stoma acceptance, and quality of life, as the cross-sectional design of the present study precludes causal inference. Second, intervention studies targeting stigma reduction and stoma acceptance enhancement—particularly among the high-stigma group—are warranted to test the clinical utility of the differentiated strategies proposed above. Third, validation of the three-profile LPA solution across diverse cultural and clinical settings (e.g., non-tertiary hospitals and different geographic regions) is necessary to assess its generalizability. Addressing these questions will help translate our preliminary findings into evidence-based, patient-centered care.

## Conclusions

In conclusion, significant inter-individual heterogeneity in stigma exists among patients with CRC and an intestinal ostomy, with three latent profiles identified: the low, moderate, and high stigma groups. Marital status, household income, and self-care ability are important predictors distinguishing the high stigma group. Furthermore, stoma acceptance shows a statistical pattern consistent with partial mediation in the relationship between different stigma profiles and quality of life. These findings provide preliminary hypotheses and target candidates for future intervention studies.

## Data Availability

The datasets used and analysed during the current study are available from the corresponding author on reasonable request.
